# Efficient Wnt mediated intestinal hyperproliferation requires the cyclin D2-CDK4/6 complex

**DOI:** 10.1186/1747-1028-6-3

**Published:** 2011-02-02

**Authors:** Kevin Myant, Owen Sansom

**Affiliations:** 1 The Beatson Institute for Cancer Research, Garscube Estate, Switchback Road, Bearsden, Glasgow, G61 1BD, UK

## Abstract

Inactivation of the gene encoding the adenomatous polyposis coli (APC) tumour suppressor protein is recognized as the key early event in the development of colorectal cancers (CRC). *Apc *loss leads to nuclear localization of beta-catenin and constitutive activity of the beta-catenin-Tcf4 transcription complex. This complex drives the expression of genes involved in cell cycle progression such as c-Myc and cyclin D2. Acute loss of *Apc *in the small intestine leads to hyperproliferation within the intestinal crypt, increased levels of apoptosis, and perturbed differentiation and migration. It has been demonstrated that c-Myc is a critical mediator of the phenotypic abnormalities that follow *Apc *loss in the intestine. As it may be difficult to pharmacologically inhibit transcription factors such as c-Myc, investigating more druggable targets of the Wnt-c-Myc pathway within the intestine may reveal potential therapeutic targets for CRC. Recent work in our laboratory has shown that the cyclin D2-cyclin-dependent kinase 4/6 (CDK4/6) complex promotes hyperproliferation in *Apc *deficient intestinal tissue and *Apc^Min/+ ^*adenomas. We showed that the hyperproliferative phenotype associated with *Apc *loss *in vivo *was partially dependent on the expression of cyclin D2. Most importantly, tumour growth and development in *Apc^Min/+ ^*mice was strongly perturbed in mice lacking cyclin D2. Furthermore, pharmacological inhibition of CDK4/6 suppressed the proliferation of adenomatous cells. This commentary discusses the significance of this work in providing evidence for the importance of the cyclin D2-CDK4/6 complex in colorectal adenoma formation. It also argues that inhibition of this complex may be an effective chemopreventative strategy in CRC.

## Introduction

Unscheduled cell division is characteristic of cancer cells [1]. As such, normal cell division is a tightly controlled process that only permits cells to divide in a timely and restricted manner. This process is directed by two classes of proteins, a group of serine/threonine kinases termed cyclin-dependent kinases (CDKs) and their activating binding partners, cyclins [2].

Heterodimeric complexes of CDKs and cyclins drive all stages of the cell cycle. The G1/S phases of the cell cycle are primarily directed by the sequential phosphorylation of the retinoblastoma susceptibility protein Rb. Hypophosphorylated Rb binds to E2F and functions as a transcriptional repressor of E2F target genes. In early G1 phase, CDK4 and CDK6 are activated by D-type cyclins leading to phosphorylation of Rb. This leads to partial dissociation of the E2F/Rb complex and expression of E2F target genes required for cell cycle progression such as cyclins A and E. The accumulation of cyclin E leads to activation of CDK2 and further phosphorylation of Rb. This leads to full release of E2F, further activation of E2F targets and entry into S phase [3,4]. During S phase a complex of cyclin A-CDK2 drives S phase progression by phosphorylation of various proteins involved in DNA replication [5]. As S phase concludes, the activity of cyclin A-CDK1 initiates prophase and finally, the cyclin B-CDK1 complex contributes to completion of mitosis [6,7]. Cell cycle progression is also controlled by inhibition of cdk activity by two families of inhibitors. Cyclin D-CDK4/6 complexes are inhibited by the INK4 family (p15^INK4b^, p16^INK4a^, p18^INK4c ^and p19^INK4d^) and cyclin A/B/E-CDK1/2 complexes by the Cip/Kip family (p21^Cip1/Waf1/Sdi1^, p27^Kip1 ^and p57^Kip2^) [8].

Given increased cellular proliferation is a key feature of tumourigenesis, it would be predicted that inappropriate activation of CDK/cyclin complexes would occur in cancer. This has proven to be the case, with deregulation of CDK4 and CDK6 implicated in a wide variety of tumours [9]. A common mechanism by which this occurs is inactivation of p16^INK4a ^by gene deletion, point mutation or promoter methylation. Alternatively, CDK4 and/or CDK6 activity can be increased and this is observed in several tumour types including glioma, breast tumours, lymphoma and melanoma [9]. Cyclin D1 is the most commonly over expressed D-type cyclin in human tumours. Its deregulated expression is observed in a wide variety of tumour types including non-small cell lung cancer and carcinomas of breast, head and neck and oesophagus [10]. Additionally, cyclin D2 is commonly over expressed in colon tumours and its over expression may be related to a higher tumour-node-metastasis (TNM) stage of tumour [11]. E-type cyclins are also frequently over expressed, and their inhibitors p21^Cip1/Waf1/Sdi1 ^and p27^Kip1 ^often silenced, in human tumours suggesting that CDK2 activity may be dysregulated in human cancer [12].

## Discussion

In a recent study from our laboratory we set out to test the requirement for the cyclin D2-CDK4/6 complex in driving cell cycle progression following activation of Wnt signalling due to *Apc *loss [13]. Previous work from our laboratory has demonstrated that loss of *Apc *in the mouse intestine leads to enhanced Wnt signalling which drives activation of c-Myc [14]. We have also shown that c-Myc is required for Wnt driven intestinal proliferation [15]. As it has been demonstrated that the cyclin D-CDK4/6 complex is an important downstream mediator of c-Myc signalling we reasoned that cyclin D2 may be required downstream of c-Myc for proliferation following loss of *Apc *[16,17]. We initially demonstrated that expression of both cyclin D2 and CDK4 are upregulated following *Apc *loss in the intestinal epithelium. Moreover, we showed that expression of this complex requires c-Myc and correlates with phosphorylation of Rb, indicating that these over expressed proteins form a functionally active complex.

To test whether cyclin D2 is required for hyperproliferation following acute Wnt activation we crossed *cyclin D2 *deficient mice to ones carrying inducible floxed alleles of *Apc*. Interestingly, when we specifically deleted *Apc *in the small intestine of these mice we observed a significant reduction in proliferation of *Apc/cyclin D2 *double deficient intestines compared to the intestines of *Apc *deficient mice. This is in contrast to normal enterocyte intestinal proliferation within the *cyclin D2 *deficient mice. This indicates that efficient proliferation in cells where Wnt signalling is deregulated requires high levels of cyclin D2, whereas normal proliferation is not cyclin D2 dependent.

*Apc^Min/+ ^*mice are heterozygous for a truncating mutation at codon 850 of *Apc*. Spontaneous loss of the wildtype copy of *Apc *leads to initiation of multiple intestinal adenomas that model early stages of CRC. Similar to acute *Apc *loss, the ademonas that arise in this model show elevated Wnt signalling and their proliferation is dependant upon over expression of c-Myc [18]. We investigated the effect of cyclin D2 loss in this model and observed a slowing of tumourigenesis and reduced proliferation in the intestinal tumours of mice lacking cyclin D2. This further demonstrates a requirement for cyclin D2 in Wnt driven hyperproliferation. Although not immediately expressed following Apc loss, cyclin D1 accumulates in the nuclei of Apc deficient tumour cells [19,20]. To test if cyclin D1 could be partially compensating for the loss of cyclin D2 in *Apc *deficient cells we combined cyclin D2 loss with cyclin D1 heterozygosity in *Apc^Min/+ ^*mice. We found that this had an additive effect on suppression of tumourigenesis with very low numbers of tumours developing in the double deficient mice. Furthermore, the tumours that did arise had very low numbers of proliferative cells. This raises the tempting possibility that inhibition of cyclin D-CDK4/6 complexes may prevent proliferation of *Apc *deficient cells.

To test this hypothesis and to confirm that the effects on proliferation mediated by cyclin deficiency were due to interactions with CDK4/6, we treated *Apc^Min/+ ^*mice that had developed tumours with a novel small-molecule pharmacologic inhibitor of CDK4/6, PD0332991. This molecule has been shown to selectively inhibit CDK4/6 *in vivo *and can initiate regression in colo-205 colon carcinoma xenografts [21,22]. Following treatment with this inhibitor for 5 days, tumours showed reduced proliferation levels, whereas normal tissue was proliferating normally. This data provides additional evidence that *Apc *deficient tissue is particularly dependant on high levels of cyclin D2-CDK4/6 activity for proliferation.

Animal knockout models of cyclin D-CDK family members have indicated a large amount of functional redundancy between members of different complexes [12]. This raises the possibility that specific inhibition of particular CDKs may not be successful or may lead to development of resistance. In our study, we demonstrated that short term treatment with PD0332991 is capable of illicting a cellular response similar to that of genetic deletion of cyclin D2. How this translates over the longer term in this model remains to be seen, and it is possible that over time other CDKs may compensate for inhibition of CDK4/6. Encouragingly, rechallenge experiments performed with Colo-205 colon xenografts showed no evidence of resistance developing after treatment with PD0332991 [22]. Thus, it may be the case that *Apc *deficient intestinal cells are exquisitely sensitive to CDK4/6 inhibition and there is no immediate redundancy between CDKs. The study we carried out focused on the initial stages of colorectal adenoma formation. A number of murine models that mimic later stages of CRC have recently been developed [23,24]. A focus of future research should be to determine the efficacy of PD0332991 and similar compounds in suppressing tumour growth in models such as these. This will give us a better understanding of the roles of cyclin/CDK complexes in later stage disease and determine if their inhibition may be a viable strategy in the treatment of CRC.

## Conclusions

**Figure 1:**
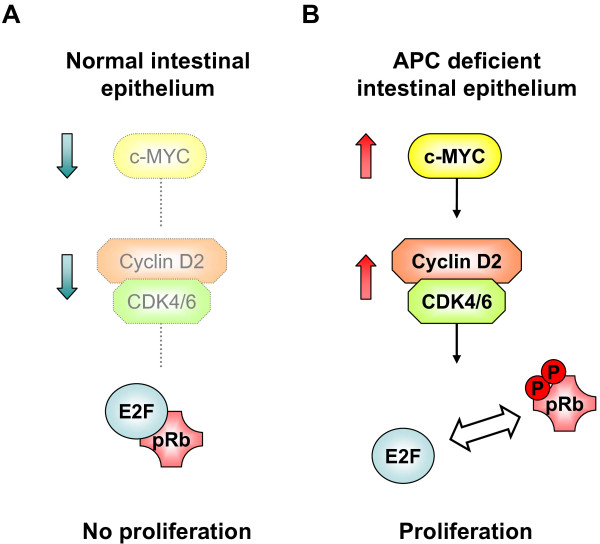
**Cyclin D2-CDK4/6 complex drives efficient hyperproliferation in *Apc *deficient cells**. (A) In the normal intestinal epithelium Wnt activity and hence c-Myc levels are restricted. This prevents accumulation of the cyclin D2-CDK4/6 complex and suppresses proliferation. (B) In *Apc *deficient cells Wnt activity and c-Myc levels are high. The high levels of c-Myc lead to accumulation of cyclin D2 and CDK4/6, possibly via their direct transcriptional activation. The complex of cyclin D2 and CDK4/6 phosphorylates Rb and permits E2F to drive cell cycle entry and subsequent hyperproliferation.

In conclusion, our study has provided important *in vivo *evidence of an essential role for the cyclin D2-CDK4/6 complex in efficient hyperproliferation in tumour cells where Wnt is activated (Figure 1). We would therefore argue that the use of CDK4/6 inhibitors such as PD0332991 warrants further investigation in murine models of CRC.

## Competing interests

The authors declare that they have no competing interests.

## Authors' contributions

KM wrote the manuscript and designed the figure. OS provided advice, support and proof read the manuscript. Both authors read and approved the final manuscript.
